# Prediction of relapse in myelin oligodendrocyte glycoprotein antibody-associated disease: external validation of the MOG-AR score

**DOI:** 10.1007/s00415-026-13754-9

**Published:** 2026-03-23

**Authors:** Wei Zhen Yeh, Anna Francis, Helmut Butzkueven, Ruth Geraldes, Maria Isabel Leite, Jacqueline Palace

**Affiliations:** 1 Nuffield Department of Clinical Neurosciences, University of Oxford, Oxford, UK; 2 Department of Clinical Neurology, John Radcliffe Hospital, Oxford University Hospitals NHS Foundation Trust, Oxford, UK; 3 Department of Neuroscience, School of Translational Medicine, Monash University, Melbourne, Australia; 4 Department of Neurology, Alfred Health, Melbourne, Australia; 5 Department of Clinical Neurology, Level 3 West Wing, John Radcliffe Hospital, Headley Way, Headington, Oxford, OX3 9DU UK

**Keywords:** Myelin oligodendrocyte glycoprotein antibody-associated disease, MOGAD, Relapse, Prediction, Risk score, Validation

## Abstract

**Background:**

Predicting relapses in myelin oligodendrocyte glycoprotein antibody-associated disease (MOGAD) when disability is relapse-dependent is crucial to guide treatment decisions including whether to treat from onset. MOG-AR, a relapse risk score, was recently developed in a Chinese cohort from disease onset. This study aimed to externally validate the MOG-AR score.

**Methods:**

MOGAD patients seen through the Oxford National NMO Service with ≥ 1-year disease duration and available data for MOG-AR score calculation (variables of age, sex, onset attack phenotype, treatment) were included. MOG-AR score and grade were calculated. Relapse occurrence at 3 years from onset was used as the primary outcome. MOG-AR performance was assessed by measures of discrimination and calibration.

**Results:**

We included 284 MOGAD patients with a 4.7-year median disease duration. Relapse occurred in 38% within 3 years of onset. Median MOG-AR score and grade were 11 (IQR 10–12) and 3 (3–3) for those who relapsed and 9 (8–11) and 3 (2–3) for those who did not. Observed proportion with relapse was 27%, 26%, 41%, and 53% for grades 1, 2, 3, and 4, respectively. Discrimination assessment by grade showed an area under the receiver operating characteristic curve of 0.58 (95% CI 0.52–0.63). Calibration assessment was consistent with overestimation of relapse probabilities.

**Conclusion:**

In a UK-MOGAD cohort, MOG-AR score showed suboptimal performance in predicting relapse over a 3-year period from onset. Further work to find clinically accessible predictive biomarkers and tools for a relapsing course would facilitate better treatment strategies near disease onset.

## Introduction

Myelin oligodendrocyte glycoprotein antibody-associated disease (MOGAD) is a recently defined inflammatory demyelinating disease of the central nervous system [1]. It is clinically characterized by attacks of mono- or polyfocal neurological deficits with incomplete recovery from attacks leading to disability accrual [2]. To avoid disability accrual, immunomodulatory treatments are empirically used to prevent relapse usually in relapsing patients. Between 30 and 50% of patients experience relapse within 4–5 years of their onset attack and so not all patients will benefit from longer-term treatment after a single onset attack [3, 4].

The ability to predict patients who are at high risk for relapse after their onset attack could assist in stratifying patients who may benefit from a more prolonged or aggressive treatment strategy. The MOG-AR score was recently developed using clinical variables with the aim of enabling relapse risk prediction [5]. However, its performance has only recently been externally validated in a different population to the one MOG-AR was developed on and studies evaluating its transportability remain limited [6–8]. In this study, we aimed to externally validate the MOG-AR score in a large MOGAD cohort from the United Kingdom (UK).

## Methods

### Study design and participants

Patients who were seen through the Oxford Neuromyelitis Optica Highly Specialised Service and provided written informed consent to participate in the Demyelinating Research Tissue Bank (approved by Oxford C Research Ethics Committee, reference 21/SC/0353) were considered for inclusion. Data of patients were prospectively collected upon entry into this service. Collected data include patient demographics, attack characteristics and immunomodulatory treatments used. Patients are reviewed by at least one of two neurology consultants (M.I.L., J.P.) with expertise in MOGAD in this service. Data were extracted in February 2025.

We included patients who fulfilled MOGAD diagnostic criteria [1], had ≥ 1-year disease duration by last follow-up, and available clinical data to calculate the MOG-AR score and outcome, specifically age, sex, onset attack phenotype, and immunomodulatory treatments used as well as relapse occurrence.

### MOG-AR score, variables and outcomes

The MOG-AR score was developed in a registry-based multicenter Chinese cohort of MOGAD patients with the aim of predicting relapse risk following disease onset [5]. MOG-AR score ranges from 0 to 16 and is then stratified into four grades (grades 1 to 4), with higher grades associated with higher relapse risks. The authors provide absolute relapse risks for each of the four grades, although the specific time horizon for these was not explicitly specified [5]. Five variables were used for calculation of the MOG-AR score: age at disease onset, sex, attack phenotype, immunosuppressive therapy use, and oral corticosteroid duration (< 3 or ≥ 3 months). Points assignation to each variable category is shown in Table 1.

**Table 1:** Cohort characteristics

		MOG-AR score points assignation	Overall cohort (*n* = 284)	Nonrelapsers (*n* = 177)	Relapsers (*n* = 107)	*p*
Age at onset, median (IQR), y		2 if ≥ 45 y; 0 if < 45 y	31.3 (16.9, 43.1)	31.3 (17.2, 41.8)	29.8 (16.4, 44.4)	0.91
Sex, *n* (%)	Female	2	169 (59.5)	104 (58.8)	65 (60.7)	0.84
	Male	0	115 (40.5)	73 (41.2)	42 (39.3)	
Onset attack phenotype, *n* (%)	ADEM	3	14 (4.9)	12 (6.8)	2 (1.9)	0.24
	Brainstem/ cerebellar deficits	1	4 (1.4)	1 (0.6)	3 (2.8)	
	Cerebral cortical encephalitis	4	1 (0.4)	1 (0.6)	0 (0)	
	Cerebral monofocal or polyfocal deficits	1	6 (2.1)	4 (2.3)	2 (1.9)	
	Mixed	2	48 (16.9)	29 (16.4)	19 (17.8)	
	Myelitis	0	46 (16.2)	32 (18.1)	14 (13.1)	
	Optic neuritis	2	165 (58.1)	98 (55.4)	67 (62.6)	
Immunosuppressive therapy for ≥ 6 months after first attack, n (%)	No	5	271 (95.4)	166 (93.8)	105 (98.1)	0.16
	Yes	0	13 (4.6)	11 (6.2)	2 (1.9)	
Oral steroid for ≥ 3 months after first attack, n (%)	No	3	154 (54.2)	73 (41.2)	81 (75.7)	< 0.001
	Yes	0	130 (45.8)	104 (58.8)	26 (24.3)	
Disease duration, median (IQR), years		–	4.7 (2.4, 8.6)	4.3 (2.4, 8.5)	5.3 (2.4, 8.6)	0.52
MOG-AR score, median (IQR)		–	10 (9, 12)	9 (8, 11)	11 (10, 12)	< 0.001
MOG-AR grade, median (IQR)		–	3 (3, 3)	3 (2, 3)	3 (3, 3)	0.006

*ADEM* acute disseminated encephalomyelitis

We generated variables required for MOG-AR score calculation. Specifically, these were age at onset ≥ 45 years, sex, onset attack phenotype, steroid-sparing immunosuppressive therapy for at least 6 months, and oral corticosteroid duration for < 3 months. Therapy variables were based on use prior to the primary outcome, i.e., first relapse.

Relapse was defined as a new clinical attack > 30 days after the prior attack. Primary outcome was relapse occurrence within three years of disease onset. As sensitivity analyses, we i) used relapse occurrence within 1 year of disease onset as the outcome, and ii) assessed the primary outcome in a sub-cohort with minimum 3-year disease duration.

### Statistical analyses

Summary statistics were used to describe cohort characteristics, and Mann–Whitney U tests and Fisher’s exact tests were used to compare characteristics between relapsers and non-relapsers. To validate MOG-AR, MOG-AR scores and grades were calculated for each patient. MOG-AR performance was assessed by the Brier score (a measure of overall performance, calculated as the mean squared difference between predicted probabilities and actual outcomes) and indices of discrimination (ability to differentiate between those who do or do not relapse) and calibration (how well predicted and observed outcomes agree). For discrimination, the area under the receiver operating characteristic curve (AUROC) was used. We defined an AUROC ≥ 0.7 as indicating fair discriminative ability. For calibration, we plotted calibration plots (plot of predicted probability vs observed outcomes) and calculated the observed/expected ratio (O/E ratio, ratio of total observed-to-expected to relapse), calibration-in-the-large (CIL, a measure which compares the mean number of predicted outcomes and the mean number of observed outcomes), and calibration slope (measure which quantifies the spread of predicted risks relative to observed outcomes) [9, 10]. Statistical analyses were conducted in the R Statistical Environment (v4.4.1) with the tidyverse and pROC packages [11–13].

## Results

### Cohort characteristics

**Fig. 1:**
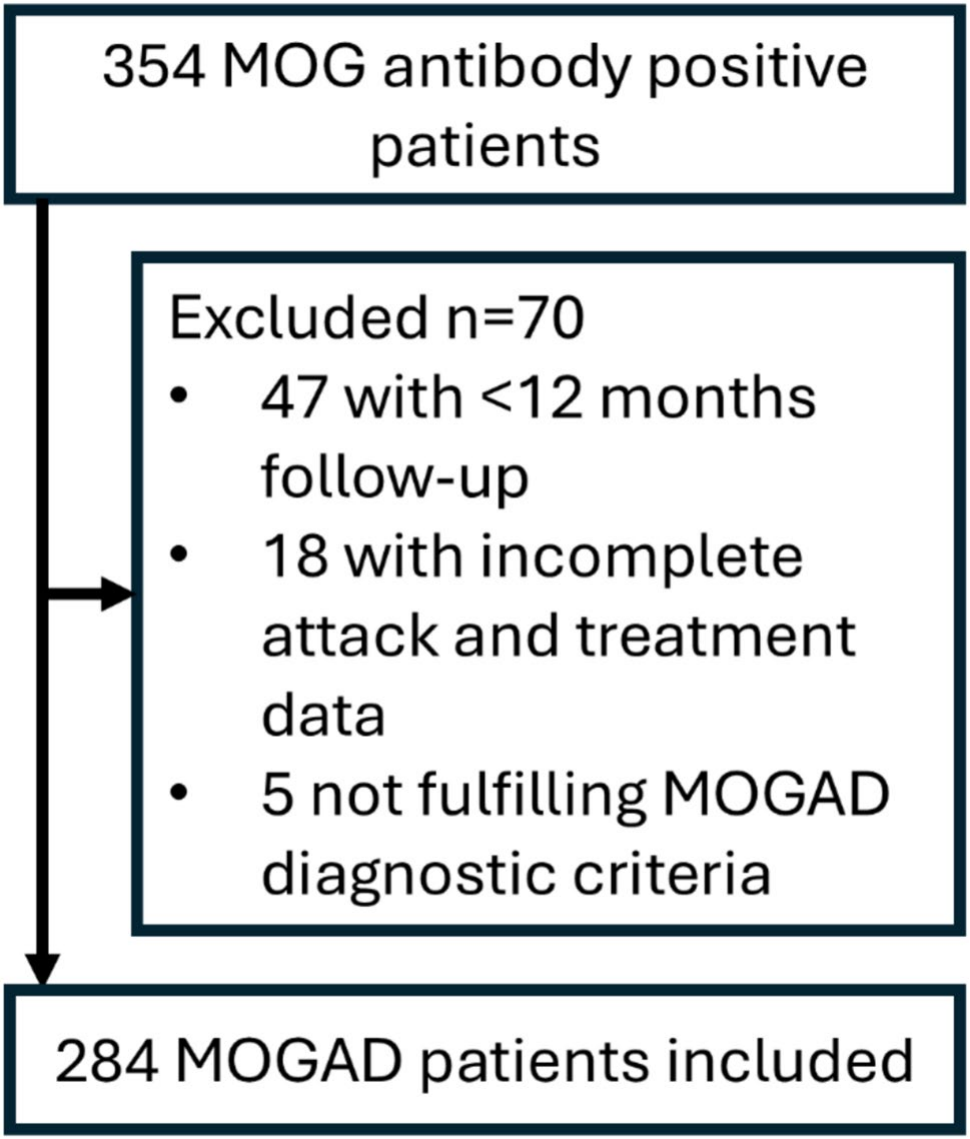
Patient inclusion flow chart. *MOG* myelin oligodendrocyte glycoprotein, *MOGAD* myelin oligodendrocyte glycoprotein antibody-associated disease

We included 284 MOGAD patients with a median disease duration of 4.7 years at last follow-up (Fig. 1). Median age at onset was 31.3 years (interquartile range [IQR] 16.9–43.1, range 1.4–77.2; Table 1) with a slight female predominance (59.5%). Isolated optic neuritis was the most common attack phenotype (58.1%).

**Fig. 2:**
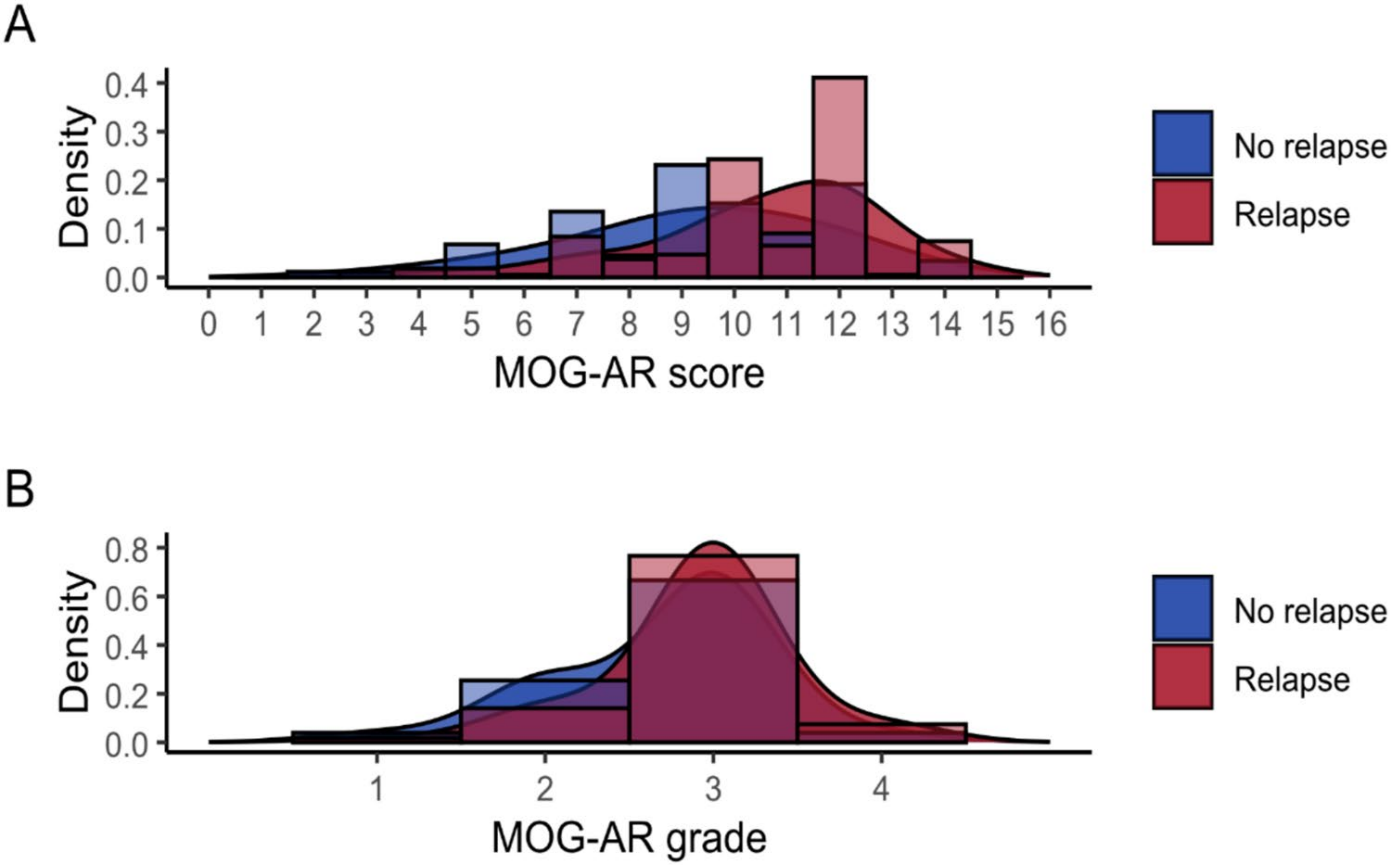
Histograms and density plots for MOG-AR score and grade by relapse occurrence within 3 years of onset attack. **A** shows MOG-AR scores and **B** shows MOG-AR grades

Relapse occurred in 107/284 (37.7%) patients within 3 years of the onset attack. Of variables included in calculation of the MOG-AR score, oral steroid use for ≥ 3 months was more frequent in nonrelapsers (104/177 [58.8%]) than relapsers (26/107 [24.3%], *p* < 0.001; Table 1). Other variables were not significantly different between relapsers and nonrelapsers. Median MOG-AR score was 11 (IQR 10–12) among those who relapsed and was 9 (IQR 8–11) in those who did not (*p* < 0.001). Converting to MOG-AR grade, median grade was 3 (IQR 3–3) in those who relapsed and was 3 (IQR 2–3) in those who did not (p = 0.006). Histogram and density plots for MOG-AR score and grade are shown in Fig. 2; scores and grades were higher in relapsers but with overlap of density plots.

### Validation of MOG-AR

**Fig. 3:**
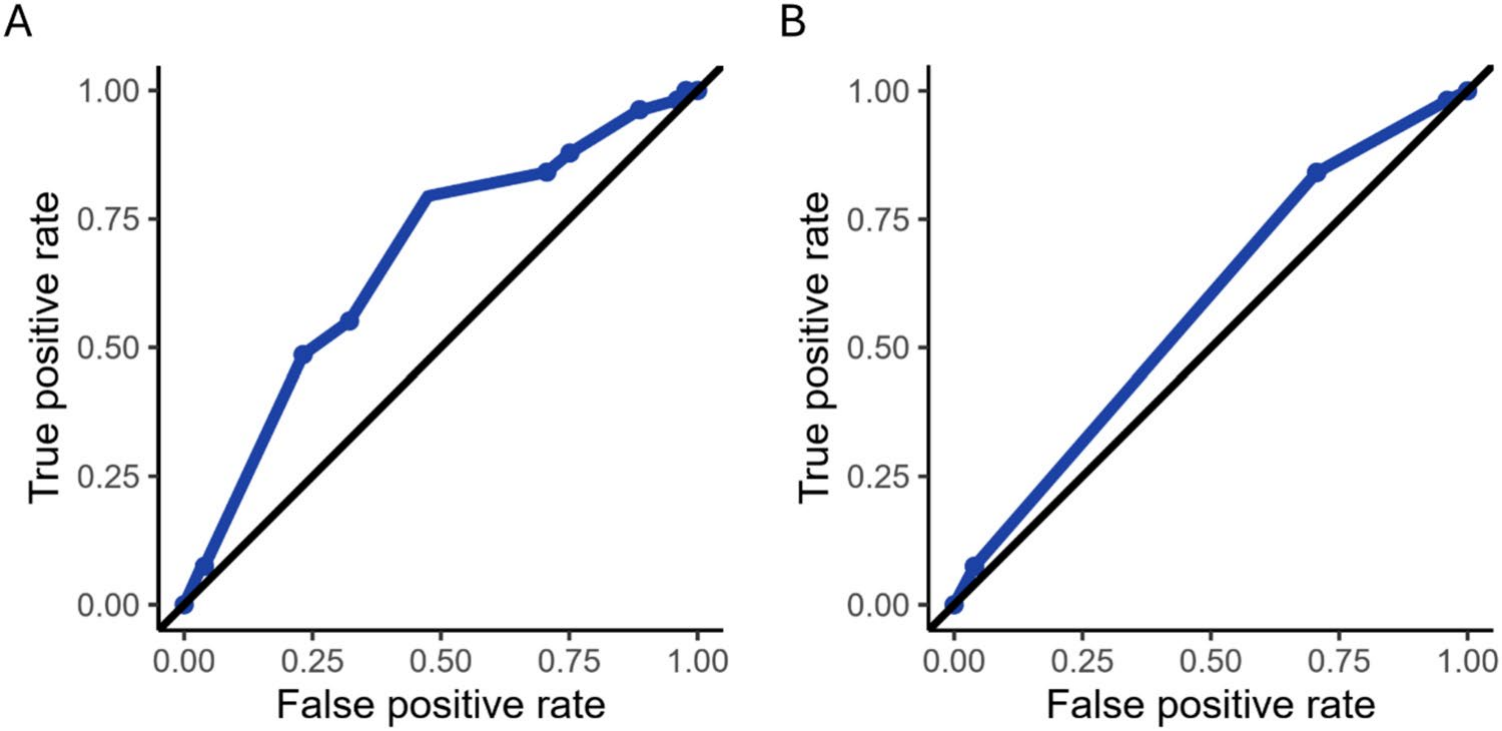
Receiver operating characteristic curves for MOG-AR score and grade in discriminating relapse occurrence within 3 years of onset attack. **A** shows the receiver operating characteristic curve for MOG-AR score and **B** shows this for MOG-AR grade

Measures for external validation are summarized in Table 2. Discrimination was assessed through calculating AUROC for MOG-AR score and grade, respectively (Fig. 3). AUROC for MOG-AR score was 0.67 (95% confidence interval [CI] 0.61–0.73) and for grade was 0.58 (95% CI 0.52–0.63), both indicating suboptimal discrimination between those who did and did not relapse within 3 years of onset.

**Table 2:** Measures of performance, calibration and discrimination of MOG-AR for 3-year relapse occurrence and sensitivity analyses

Measure	Result (95% CI) for primary outcome of three-year relapse occurrence	Result (95% CI) for outcome as one-year relapse occurrence	Result (95% CI) for outcome as three-year relapse occurrence in sub-cohort with at least three years disease duration	Interpretation
Brier score	0.30 (0.28, 0.32)	0.34 (0.32, 0.36)	0.31 (0.28, 0.34)	Measure of overall performance. Brier score ranges between 0–1, with lower scores indicating better accuracy. A “fence-sitter” (i.e., predicted probability for outcome of 0.5 for all) will lead to a Brier score of 0.25. A score of 0.30 indicates overall predictions worse than fence-sitting
Observed-to-expected ratio	0.59 (0.51, 0.68)	0.39 (0.31, 0.46)	0.58 (0.47, 0.68)	Measure of calibration. Ideal value is 1. Observed-to-expected ratio < 1 indicates over-prediction of relapse probability
Calibration-in-the-large	-1.15 (-1.40, -0.91)	-1.79 (-2.08, -1.52)	-1.18 (-1.49, -0.88)	Measure of overall calibration. Ideal value is 0. Negative values suggest overestimation of relapse probability
Calibration slope	0.66 (0.19, 1.16)	0.71 (0.15, 1.33)	0.43 (-0.14, 1.04)	Measure of spread of predicted probabilities compared to observed outcomes. Ideal value is 1. A slope < 1 indicates relapse predictions are too extreme
Area under the receiver operating characteristic curve for MOG-AR score	0.67 (0.61, 0.73)	0.66 (0.59, 0.72)	0.64 (0.56, 0.72)	Measure of discrimination. Area under the curve > 0.7 is considered to indicate acceptable discriminative ability. An area under the curve < 0.7 indicates suboptimal discrimination between those who relapse and do not relapse
Area under the receiver operating characteristic curve for MOG-AR grade	0.58 (0.52, 0.63)	0.58 (0.52, 0.63)	0.55 (0.48, 0.62)

**Fig. 4:**
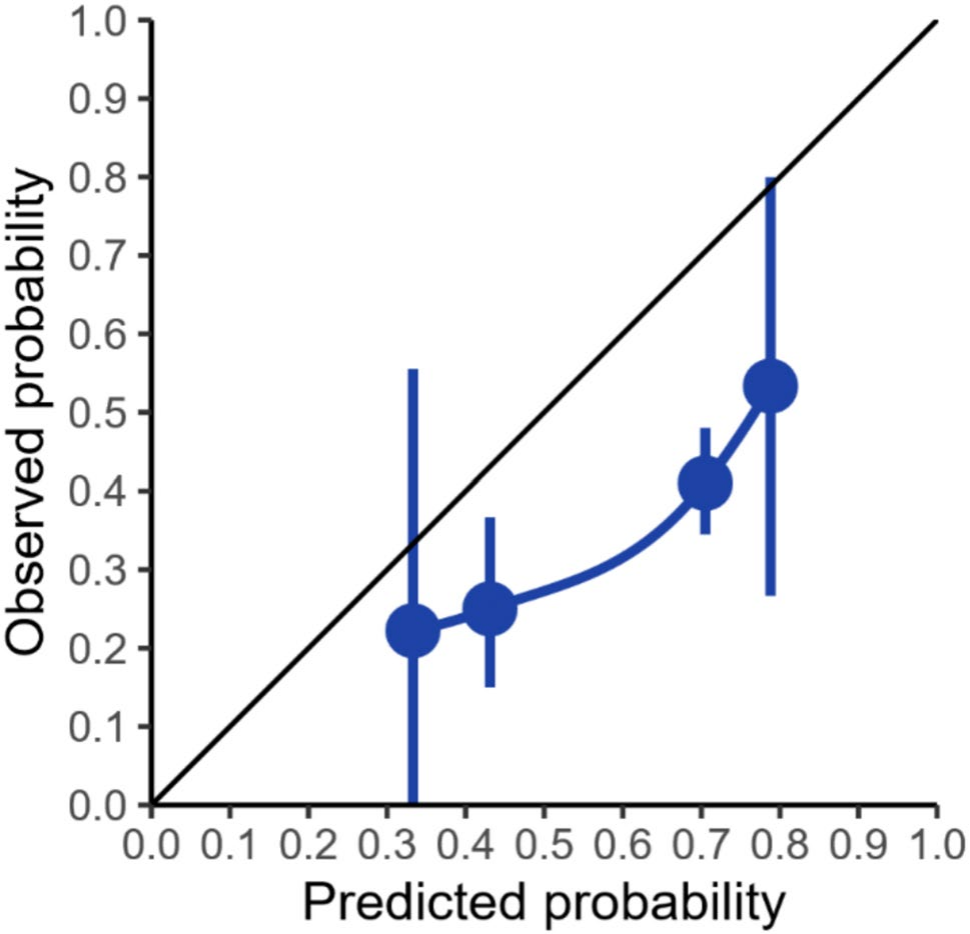
Calibration plot for MOG-AR grade and relapse occurrence within 3 years of onset attack. Error bars represent 95% confidence intervals

Assessing calibration, the calibration plot of observed by predicted probabilities for relapse for each MOG-AR grade showed overestimation of relapse risks across all grades (Fig. 4). This is consistent with the O/E ratio of 0.59 (95% CI 0.51–0.68) and CIL of −1.15 (95% CI −1.40–−0.91), both indicating overprediction of relapse probabilities (Table 2). Calibration slope was < 1, which in combination with the calibration plot, indicated predicted relapse probabilities were too extreme particularly at higher grades (Table 2, Fig. 4).

### Sensitivity analyses

Although the time horizon of relapse in the development of MOG-AR was not explicitly stated, the authors discussed that it could be used to help assess risk within 1 year of onset [5]. We, therefore, assessed 1-year relapse occurrence in a sensitivity analysis. Results were consistent with our primary analyses, showing suboptimal performance and discriminative ability, and overestimation of relapse risks (Table 2). In our second sensitivity analysis in a sub-cohort with at least 3-year disease duration by last follow-up, results were also consistent with the primary analyses (Table 2).

## Discussion

In our external validation study of MOG-AR, MOG-AR did not perform well as a risk score in the prediction of relapse occurrence within three years of MOGAD onset in our UK cohort. Discrimination of relapsing status by MOG-AR score and grade were both suboptimal (AUROC < 0.7) and calibration assessment showed overestimation of relapse probabilities. Sensitivity analyses showed consistent results.

External validation of a risk score or prediction model is important to assess its generalizability, transportability, and clinical utility [9]. Internal validation of MOG-AR by random split reported an AUROC of 0.709 for discrimination of relapsing status by MOG-AR score [5]. However, a random split approach is not recommended in model development and internal validation particularly for cohorts of small sample size. In that scenario, it can lead to insufficient sample size for adequate model development with resultant overfitting. Model performance can be overestimated when validated in the same cohort. In our external validation study, AUROC for both MOG-AR score and grade were both < 0.7, indicating inadequate discriminative ability in our United Kingdom cohort.

There are several possible reasons for an inadequate performance of MOG-AR in our cohort. First, differences in population race and patient characteristics can contribute. There may be a referral bias to specialist centers favoring relapsing patients in the cohort used to develop MOG-AR. Our cohort is from one of two highly specialized UK MOGAD services where most patients diagnosed with this condition in the UK will be reviewed in. Despite this, our sensitivity analysis of patients with a minimum of 3 years’ disease duration—which is relatively more enriched for relapsing patients than our overall cohort as they are less likely to be lost to follow-up—failed to validate MOG-AR as an effective prediction score. Second, development using a recurrent event model may not be appropriate to apply to predicting relapse from onset. Although the authors of MOG-AR propose application to predict relapse early in the course of MOGAD to guide treatment decisions, the baseline hazard and predicted risks of a first relapse after onset may be biased from an Andersen–Gill model. Third, split sample of a limited cohort for development and internal validation can lead to overfitting of the model, with poorer performance on external validation. Fourth, although the authors of MOG-AR provide predicted relapse risks for each grade, the specific time horizon they used to calculate these was not specified in their paper [5]. This is important for contextualization of predicted relapse probabilities, and is recommended to be reported as per prediction model reporting guidelines [14]. We assessed relapse occurrence over three years—which is a longer duration than the median disease duration at last follow-up in the derivation cohort—but were still unable to validate MOG-AR.

One recent study validated MOG-AR in an independent Chinese cohort and reported reasonable discriminative ability for relapse within three years from onset with an AUROC of 0.72 for MOG-AR score, although the lower limit of the 95% CI was < 0.7 [8]. Sample size was also limited in that study at 157 patients. Their cohort was from a tertiary center and included patients of Chinese ethnicity, with their patient characteristics likely similar compared to that used for derivation of MOG-AR. Conversely, MOG-AR did not perform well in our cohort, potentially indicating poor transportability. Our study was concordant with two recent external validation studies which assessed MOG-AR in an Italian and North American population [6, 7], respectively. Both showed suboptimal discrimination between those who relapsed and those who did not, with reported AUROC of 0.64 and 0.48 by these respective studies.

Studies of clinical predictors for relapse have not identified factors with consistent prognostic value. In MOG-AR, onset age ≥ 45 years was associated with elevated relapse risk [5]. However, a study of an independent Chinese cohort did not show an association between age and relapse risk using the same cut-point of 45 years [15]. Other studies in European predominant populations had found young adults (18–39 years) to have a higher relapse risk than other age groups [3, 4]. Evidence for sex as a risk factor has been mixed. Female sex was found to associate with elevated relapse risk in the MOG-AR model and several additional studies [4, 16], but not in others [3, 15, 17–20]. In regard to onset attack phenotype, the derivation model for MOG-AR identified cerebral cortical encephalitis to associate with the highest relapse risk compared to other phenotypes [5]. However, this is a rare phenotype in an already small sample, and therefore a very unreliable result. In another study, optic neuritis onset had a higher relapse probability than myelitis at onset, although this result was not robust to adjusting for steroid treatment [3]. Other studies did not identify a significant difference in relapse risk between phenotypes at onset [4, 15]. Steroid and steroid-sparing immunosuppressant use were included in the MOG-AR development model as fixed variables [5]. MOGAD is well-recognized to be steroid responsive [21, 22]. Apart from the potential for biased estimates due to immortal time bias (as patients will need to have survived and not had a relapse for a minimum duration in order to be classified as steroid- or immunosuppressant-exposed prior to the relapse outcome) [23], a risk score whose purpose is to predict relapse risk and guide treatment strategy should ideally include only baseline variables available at the time of decision-making [24]. Treatment variables violate this principle, particularly if the risk score is designed for prediction near onset, i.e., prior to treatment initiation. Further work is required to identify better predictors for relapse risk. Environmental and modifiable factors such as smoking may influence relapse risk and warrant further study [25]. Myelin oligodendrocyte glycoprotein antibody status and binding characteristics could have potential clinical utility in stratifying relapse risk [26–29]. Additional work to validate their predictive performance, and also to improve accessibility to such testing, will be important before these can be recommended for routine use in clinical practice.

There are several strengths to our study. We included a relatively large single specialist service cohort from one of two highly specialized services for MOGAD in the UK. Our cohort is well-phenotyped with all patient reviews conducted by one of two neurologists (J.P. and M.I.L.) with expertise in MOGAD. In terms of limitations, a larger sample size would have allowed for more precise outcome measures and particularly assessment in different racial groups, although we were still able to confidently show sub-optimal performance across most measures with our current cohort.

## Conclusion

Our external validation study of the MOG-AR score showed suboptimal performance for relapse prediction after onset attack in our UK MOGAD cohort. Identifying reliable predictors of relapse risk in MOGAD remains an important goal to stratify treatment strategies near MOGAD disease onset.

## Data Availability

The anonymized grouped data that support the findings of this study are available from the corresponding author to qualified noncommercial investigators, on reasonable request and in accordance with consent and ethics requirements.
